# Periodontitis

**Published:** 1870-12

**Authors:** W. H. Waite

**Affiliations:** Liverpool, England.


### ARTICLE IX.

### *Periodontitis.*

By W. H. Waite, D. D. S., Liverpool, England.

The disorder known by the above name consists in an inflamed condition of the investing membrane of the roots of teeth. It may occur on one root only, and in one spot, very circumscribed, or be found extensively spread over nearly the whole surface of the roots. Accompanying this inflammatory state, there is frequently a thickening of the peridental membrane, causing more or less protrusion of the tooth from the socket. The symptoms are tolerably defined, and the diagnosis easy. If the opposing tooth is *in situ*, pain is experienced in striking the jaws together; while at the same time there is present a desire to grind the teeth, for by so doing a kind of temporary relief is afforded. A gnawing, boring pain, considerably aggravated when the head is laid down, and warm at night—not quite so acute as true toothache (except in the worst stages) but quite as distressing—having a marked effect on the mental energies, some degree of concomitant inflammation about the gums in the neighborhood, and soreness to the touch—these are the chief features arising in connection with this disease.

Sometimes a patient will be unable to locate the sensations with sufficient precision to indicate the offender, and in such cases, if the teeth are gently tapped, one at a time, with the handle of an excavator, the defaulter will not fail to respond. The causes of this affection enumerated in some of our standard works are various, and, without doubt, periodontitis may proceed from numerous predisposing and exciting causes; still, probably, it would not be unsafe to say that fully 95 *per cent.* of these cases are attributable either to the presence of a dead pulp, or to the manipulation and treatment employed for the devitalization and extirpation of the pulp. The worst forms of periodontitis are to be found where, the cavity of decay having extended very near to the pulp cavity without actually exposing that organ, a large gold or amalgam filling has been inserted, and by reason of the conduction of thermal sensations through the plug, the pulp has sooner or later lost its vitality. Now, such death of pulp is speedily followed by the liberation of a foul and irritating gas, which, being confined by the filling, works its way outward through the apex of the nerve canals, and coming into contact with the periosteum, acts as an irritant thereon, inducing determination, congestion, and at length inflammation. Further on the periosteum becomes detached from the root, suppuration commences, and we have as the issue *Alveolar Abscess.*

But seeing that these large fillings of metal alone are apt to produce such unpleasant consequences, we are led to consider the advisability of interposing some non-conductor between the metal and the base of the cavity. Thus, if a gold filling be intended, we may place a layer of oxy-chloride of zinc, or Hill's Stopping, over the floor; or if an amalgam filling is to be introduced the oxy-chloride answers our purpose, and in some such way it seems probable that many large cavities among the ill-fated first molars may not only be filled but filled with a fair prospect of permanent success. Beyond a doubt, caution and the studious adoption of every preventive measure, are not thrown away in any case of deep-seated caries, for whatever may be the merits of the treatments noted hereafter, it is certainly very annoying to have a patient return shortly after the insertion of a large filling, complaining that "this tooth is so tender, I can't bear to close my mouth."

Remedies as varied as the causes of periodontitis have been suggested from time to time, most of them valuable in some circumstances, and valueless in others. It would seem as though a special bond of sympathy were called into existence between the local affection and the general health of the patient. Cold, disorder of the stomach or bowels, indigestion, rheumatic or gouty tendencies, any and every derangement of the system, appears to act, and be reacted upon by this disease; and oftentimes, baffled by the complications, and besought by the patient, we are led to give it up and remove the offending tooth, rather than encounter the apparently endless annoyance.

Happily the extreme cases are not a large majority. If "dead pulp" can be clearly diagnosed, (and the history of the case, taken together with the opacity of the tooth's appearance, will generally afford decisive evidence) and if the case presents early, say, within two or three days of the commencement of uneasy sensation, there is no quicker or surer method of procuring relief than to remove the filling, open freely into the pulp cavity, and afford a vent for the gas pent up therein, thoroughly cleansing the canals from every particle of dead tissue, and treating with dressing of camphorated spirit, followed by dilute carbolic acid, &c., till all offensive odour has disappeared, and finally when the condition of health is fully established, filling the canals, and refilling the outer cavity; or, if from any reason it be considered undesirable to remove the filling (and this may often be the case) an entrance can be made from the neck of the tooth, just at the margin of the gum, into the pulp cavity, by drilling right in through the bone, with a sharp spear-pointed drill. This is known technically as "odontrypy." Drills may be easily made of broken excavators, suitable for this purpose, and, if the tactile sense of the operator is sufficiently acute, he will observe that just as the instrument punctures the pulp-chamber, it will be felt to stick, and by this he will know that the object is accomplished. Frequently, moreover, on enquiry it will be found that the patient has tasted the peculiarly unpleasant gas thus let out into his mouth. As a rule the symptoms rapidly subside after the gas has escaped. Complete restoration may be facilitated by placing a guard of gutta-percha on the teeth of the opposite side of the head, to prevent closure of the jaws upon the affected tooth, and so shield it from irritation of that sort; also, by carefully drying the gums, and then painting the part with the official Tincture of Iodine, or the mixture given below. When periodontitis supervenes on devitalization and thorough extirpation, palliative treatment is indicated, such as free lancing of the gums, copious bleeding, after which cold applications, without and within the mouth, liberal use of Iodine, or the Iodine and Aconite, which last is certainly an excellent remedy. Again, it has been suggested that *Mercurius Vivus*, the third decimal trituration, given in small doses two or three times a day is a specific in such case. This is also a good medicine, but scarcely infallible. Indeed it is more than doubtful if anything is absolutely certain. The wise practitioner, however, is ever ready to employ any means which the peculiarities of the case, or the experience of his fellows, may suggest; and in this, as in all other affections, will not be bound by prejudice simply to follow a beaten track, but be ready on any occasion to exercise his ingenuity to discover the best treatment for each case as it presents, always bearing in mind the intimate relation that exists between this disorder and nearly every phase of systematic trouble.

Perhaps one of the greatest evils associated with this form of dental disease is the fact that patients do not generally recognize the true cause of their trouble. Neuralgia, or *tic*, as they call it in some places, seems to be a sort of universal scapegoat, on whose back are laid all kinds of dental difficulties, and to get rid of which the patients will swallow any amount of physic before they wake up to realize wherein the mischief truly consists, and then too often the disease has passed into the suppurative stage. The last hint as to the treatment of periodontitis, therefore, is never to fail in warning a patient of the possible result in every case, where there appears any reason to suppose that sooner or later the disorder may appear.—*Canada Journal of Dental Science.*

---

